# Spontaneous iliac vein rupture showed by femoral contrast bolus: A case report

**DOI:** 10.1016/j.radcr.2022.10.007

**Published:** 2022-10-31

**Authors:** Alexander A.J. Grüter, Sytse F. Oudkerk

**Affiliations:** a Department of Surgery, Amsterdam UMC, Location VUmc, Amsterdam, The Netherlands; b Department of Radiology, Noordwest Hospital, Alkmaar, The Netherlands

**Keywords:** Spontaneous iliac vein rupture, Femoral contrast bolus, CT venography, Endovascular repair, Case report, SIVR, spontaneous iliac vein rupture, CT, computed tomography

## Abstract

*Introduction:* Spontaneous iliac vein rupture (SIVR) is an uncommon disease with less than sixty cases reported before. This disease often requires surgical intervention. SIVR has never been imaged in the literature. This report shows how to image this diagnosis. *Case report:* A 71-year-old female was diagnosed with SIVR with the use of CT venography. Endovascular repair with 2 endografts and a sinus XL stent was performed. Postoperatively, the patient developed abdominal compartment syndrome and a large part of the intestines had to be removed because of ischemia. *Discussion:* This is the first report that shows SIVR before and after endovascular treatment with the use of CT venography by injecting a contrast bolus in the femoral vein. This information is of high interest for a broad range of clinicians to show or exclude a venous abdominal bleeding in an early stage.

## Introduction

Spontaneous iliac vein rupture (SIVR) is a rare disease associated with high mortality. Until now, less than 60 cases having been reported in literature since the first reported case in the 1960′s [1], [2], [3], [4]. Most of the cases are concerned of the left iliac femoral vein system and occur mainly in women [5]. The predominant left side of the disease is probably related to May-Thurner syndrome [6]. Surgical intervention is needed in the majority of the cases. So far, only 2 patients received endovascular treatment [7,8]. This disease has never been imaged in a study before. In our experience, showing how to image this disease could help many clinicians to ex- or include SIVR in an early stage. In this case report, we will present an interesting case of SIVR accompanied by CT images and videos before and after endovascular treatment.

This study was conducted in accordance with the modified PROCESS criteria, which provides a structure for reporting surgical case series [9].

## Case report

**Fig. 1:**
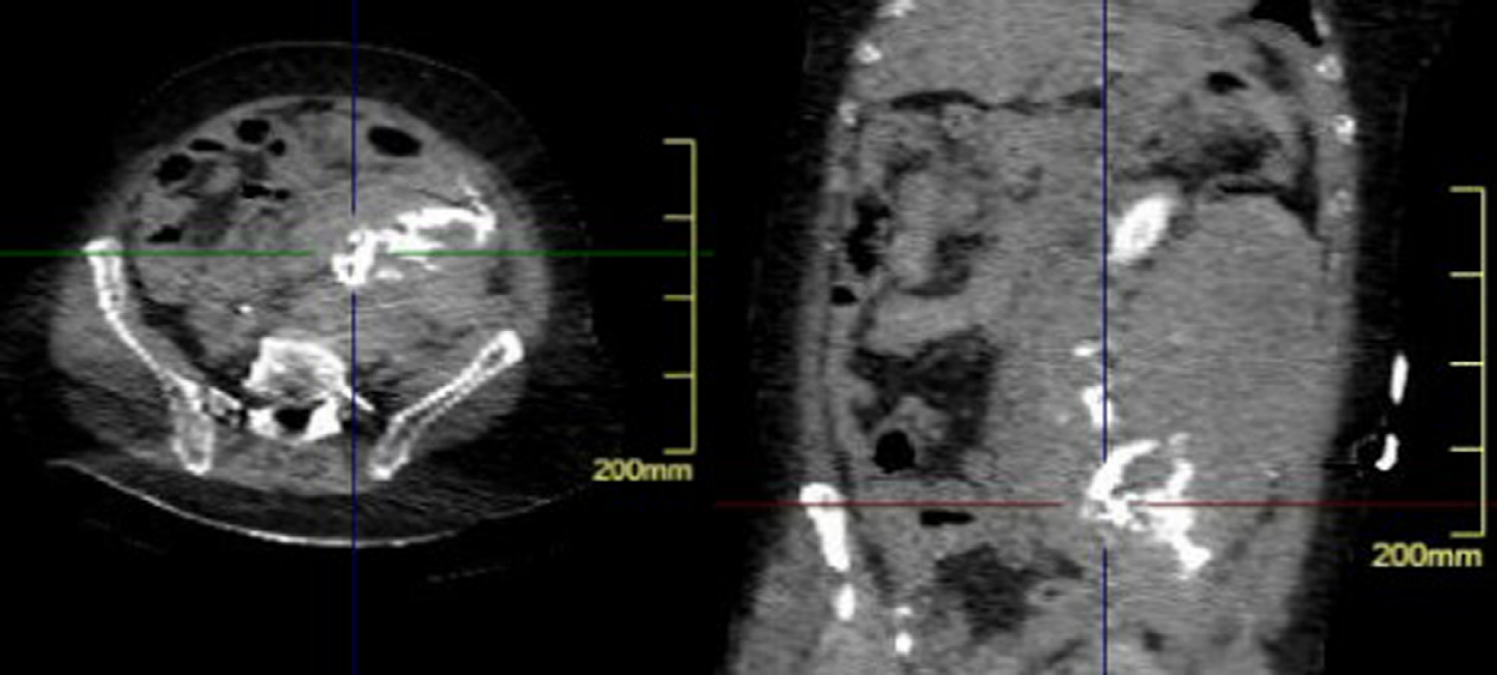
Axial and coronal view of CT scan with contrast bolus through left common femoral vein shows iliac vein rupture with contrast leakage retroperitoneal space. Notice the wide external iliac vein.

**Fig. 2:**
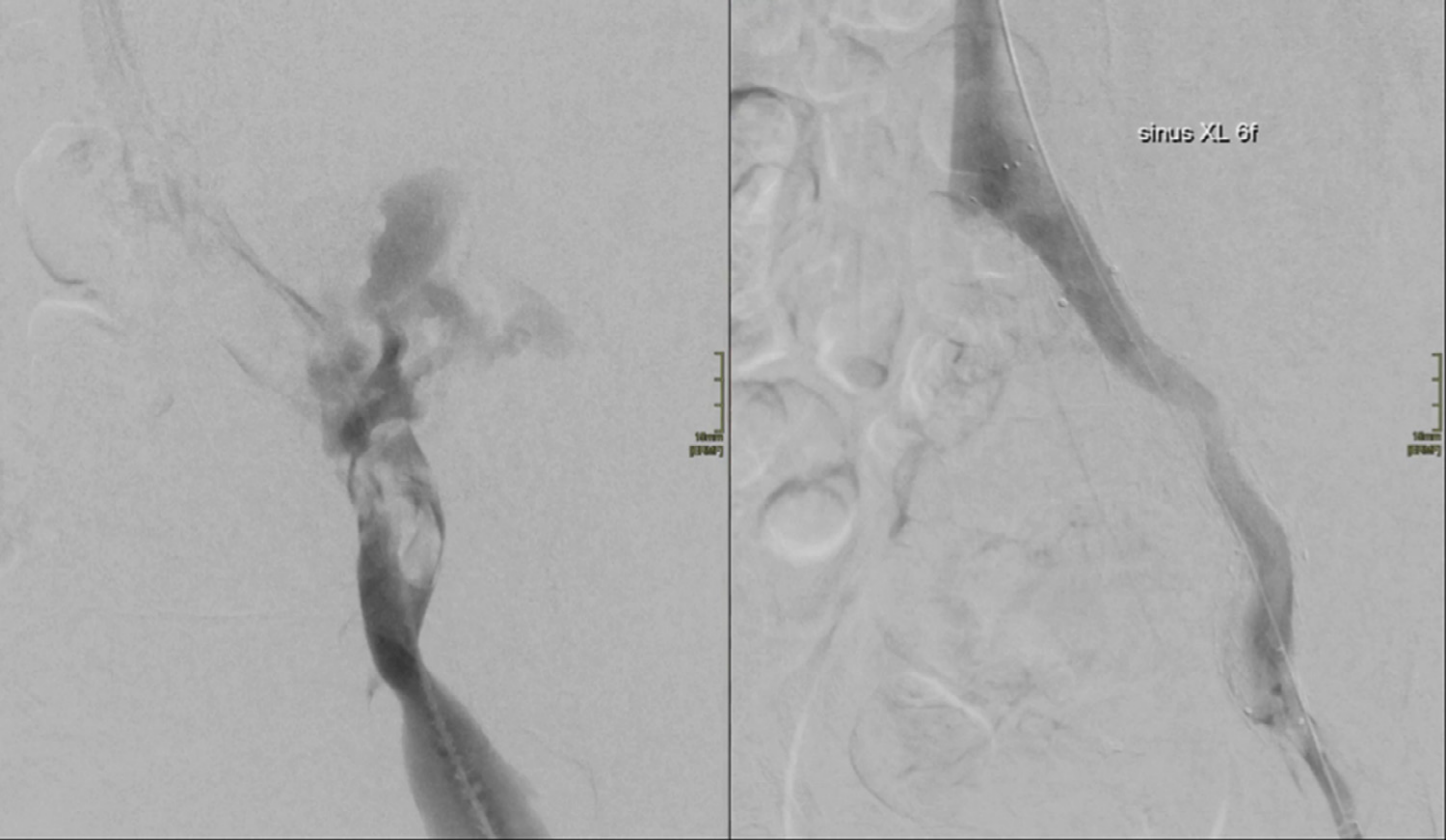
Angiography before (left) and after (right) endovascular repair.

A 71-year-old female presented to the emergency department with hypovolemic shock. Earlier that day she had acute pain in her left hip and lower part of her abdomen. She had no history of trauma. A contrast-enhanced computed tomography (CT) was obtained. This revealed a retroperitoneal hematoma on the left side, see video 1. For many hours, the diagnosis was unclear. There was no evidence of active contrast leakage on CT, but an ectatic external iliac vein was observed. Then a central venous line was put into the left femoral vein. A bolus of 50cc contrast (iodine) was injected to make a CT venography. This showed SIVR (Fig. 1 and video 2). Endovascular repair with 2 endografts and a sinus XL stent in the vein was performed (Fig. 2 and video 3). After this intervention, the patient developed abdominal compartment syndrome and because of that a laparotomy was done to remove all the retro- and intraperitoneal blood (5 liters). One day later, a left hemicolectomy was done because of a partially ischemic colon. Eventually the patient developed a complete ischemic colon and a partially ischemic small intestine. A new operation was performed to remove the ischemic parts of the intestines. Only 110 cm of the small intestine was left and an end ileostomy was performed. Eight days later, the last 15 cm of the small intestine was removed because it was ischemic. Two days after the last intervention, the patient passed away. A surgical option was not helpful anymore.

## Discussion

SIVR is a very uncommon event. To our knowledge this is the first time SIVR has been diagnosed with CT venography by injecting a contrast bolus in the femoral vein. Not to mention the fact that this has never been imaged in the literature after endovascular repair. In this case, the diagnosis was unclear for a relative long period. This report is of high relevance for a broad range of clinicians due to the fact that this imaging technique can be used in daily practice to early exclude or show a venous abdominal bleeding.

In conclusion, CT venography by injecting a contrast of bolus in the femoral vein makes it possible for early diagnosis of SIVR. This imaging technique is especially useful when a normal CT-scan shows a retroperitoneal hematoma.

## Sources of funding

This research did not receive any specific grant from funding agencies in the public, commercial or not-for-profit sectors.

## Ethics approval

Waived by the ethical committee of Amsterdam UMC, location VUmc.

## Author contribution

A.A.J. Grüter wrote the paper. S.F. Oudkerk gave his feedback on the paper and performed the procedure and the CT venography.

## Patient consent

Patient signed informed consent for the use of clinical data and images and videos of CT scan. The authors confirm that informed consent of the patient for publication has been obtained. Appropriate consent has been obtained to include case details and medical images.

## Appendix Supplementary materials

Video 1 CT scan during the arterial phase showing absence of extravasation. Image, video 1

Video 2 CT with contrast bolus through left common femoral vein shows iliac vein rupture with contrast leakage retroperitoneal space. Notice the wide external iliac vein. Image, video 2

Video 3 Angiography before (left) and after (right) endovascular repair. Image, video 3
